# Dual Mycobacterial Infection: Rare Coinfection of Mycobacterium tuberculosis and Nontuberculous Mycobacteria

**DOI:** 10.7759/cureus.100932

**Published:** 2026-01-06

**Authors:** Yubraj Aryal, Nisha K Sapkota, Tutul Chowdhury, Samuel Sule-Saa, Elliott Bondi

**Affiliations:** 1 Medicine, Interfaith Medical Center, New York, USA; 2 Pulmonary and Critical Care Medicine, Interfaith Medical Center, New York, USA; 3 Pulmonary and Critical Care Medicine, Brookdale University Hospital Medical Center, New York, USA; 4 Internal Medicine, Interfaith Medical Center, New York, USA; 5 Pulmonary and Critical Care Medicine, One Brooklyn Health, New York, USA

**Keywords:** coinfection, colonization, diagnostic challenges, mycobacterium tuberculosis, nontuberculous mycobacteria, pulmonary tuberculosis

## Abstract

There is little research on the subject, despite the rising prevalence of concurrent *Mycobacterium tuberculosis* (MTB) and nontuberculous mycobacteria (NTM) infections. We present the case of a 48-year-old immunocompetent man diagnosed with pulmonary tuberculosis with simultaneous sputum cultures positive for NTM. Chest imaging revealed bilateral upper-lobe cavitary lesions, and serial sputum studies demonstrated persistent MTB on acid-fast bacilli smears and MTB polymerase chain reaction. Standard four-drug antituberculous therapy was initiated. However, concurrent sputum cultures subsequently grew *Mycobacterium abscessus* and later *Mycobacterium avium* complex, prompting the addition of amikacin, cefoxitin, and azithromycin based on infectious disease consultation. This case illustrates the diagnostic and therapeutic challenges inherent to MTB-NTM coinfection, where distinguishing true NTM disease from colonization is critical, and underscores the importance of multidisciplinary management.

## Introduction

Tuberculosis (TB), caused by *Mycobacterium tuberculosis* (MTB), remains a major global cause of morbidity and mortality [1]. In contrast, nontuberculous mycobacteria (NTM), environmental organisms such as *Mycobacterium avium* complex (MAC), *Mycobacterium abscessus*, and *Mycobacterium kansasii, *are increasingly recognized as important pulmonary pathogens [2,3]. Although both MTB and NTM can produce chronic pulmonary disease characterized by cough, weight loss, fever, and cavitary lung lesions, they differ substantially in epidemiology and management. MTB is transmitted via human-to-human respiratory spread, whereas NTM infections arise from environmental exposure [3]. Among NTM, MAC and M. abscessus are the most common causes of clinically significant lung disease [3]. Diagnosis can be challenging because both MTB and NTM are acid-fast organisms and may produce positive sputum smears. While standard TB therapy (isoniazid, rifampin, pyrazinamide, and ethambutol) is effective for drug-susceptible MTB, NTM infections often require alternative regimens, typically macrolides, aminoglycosides, and other agents, and prolonged, species-specific treatment [1,3]. Coinfection with MTB and NTM is relatively uncommon but increasingly reported, including in immunocompetent individuals [1,2]. Although many cases are associated with underlying lung disease or immunosuppression, coinfection can occur in patients without identifiable risk factors. Distinguishing NTM colonization from clinically meaningful infection requires integration of clinical presentation, radiologic findings, and microbiologic data. Here, we describe a case of pulmonary MTB with concomitant M. abscessus and MAC infection, emphasizing the diagnostic evaluation, imaging findings, and need for tailored multidrug therapy.

## Case presentation

A 48-year-old previously healthy man presented with a three-month history of productive cough, fever at night, and systemic symptoms such as malaise and fatigue. He described white sputum production, fatigue, decreased appetite, weight loss (~5 kg), chills, and exertional dyspnea. He denied hemoptysis, pleuritic chest pain, recent travel, or known sick contacts. His only risk factor was a 10-pack-year smoking history; he had no history of immunosuppression. On exam, he was afebrile with normal vital signs except mild tachypnea with a respiratory rate of 20 per minute (Table 1). Blood pressure was 130/70 mmHg, pulse 68 per minute, and temperature 98.4°F. Lung exam revealed diffuse bilateral wheezing and crackles with reduced breath sounds; no clubbing or skin lesions were noted.

**Table 1:** Laboratory findings on admission PCR, polymerase chain reaction

Investigation	Value	Reference range
Hemoglobin	7.6	11.0-15.0 g/dL
Hematocrit	21.8	35-46%
White Blood Cell	4.5	3.8-5.3x10^6^/uL
Platelets	412	130-400x10^3^/uL
Glucose	84	80-115 mg/dL
Blood Urea Nitrogen	10	9.8-20.1 mg/dL
Creatinine	0.8	0.57-1.11 mg/dL
Sodium	131	136-145 mmol/L
Potassium	3.8	3.5-5.1 mmol/L
Chloride	100	98-107 mmol/L
Bicarbonate	30	23-31 mmol/L
Calcium	9.9	8.8-10.0 mg/dL
Albumin	4.1	3.2-4.6 g/dL
Magnesium	1.9	1.6-2.6 mg/dL
COVID-19 PCR	Negative	Negative
Prothrombin Time	12.2	9.8-13.4 sec
International Normalized Ratio	1.1	0.85-1.15
Partial Thromboplastin Time	32.2	24.9-35.9 sec
Thyroid-Stimulating Hormone	0.815	0.465-4.680 uIU/mL
T4	1.02	0.78-2.19 ng/dL
Urine Toxicology	Negative	Negative

**Figure 1:**
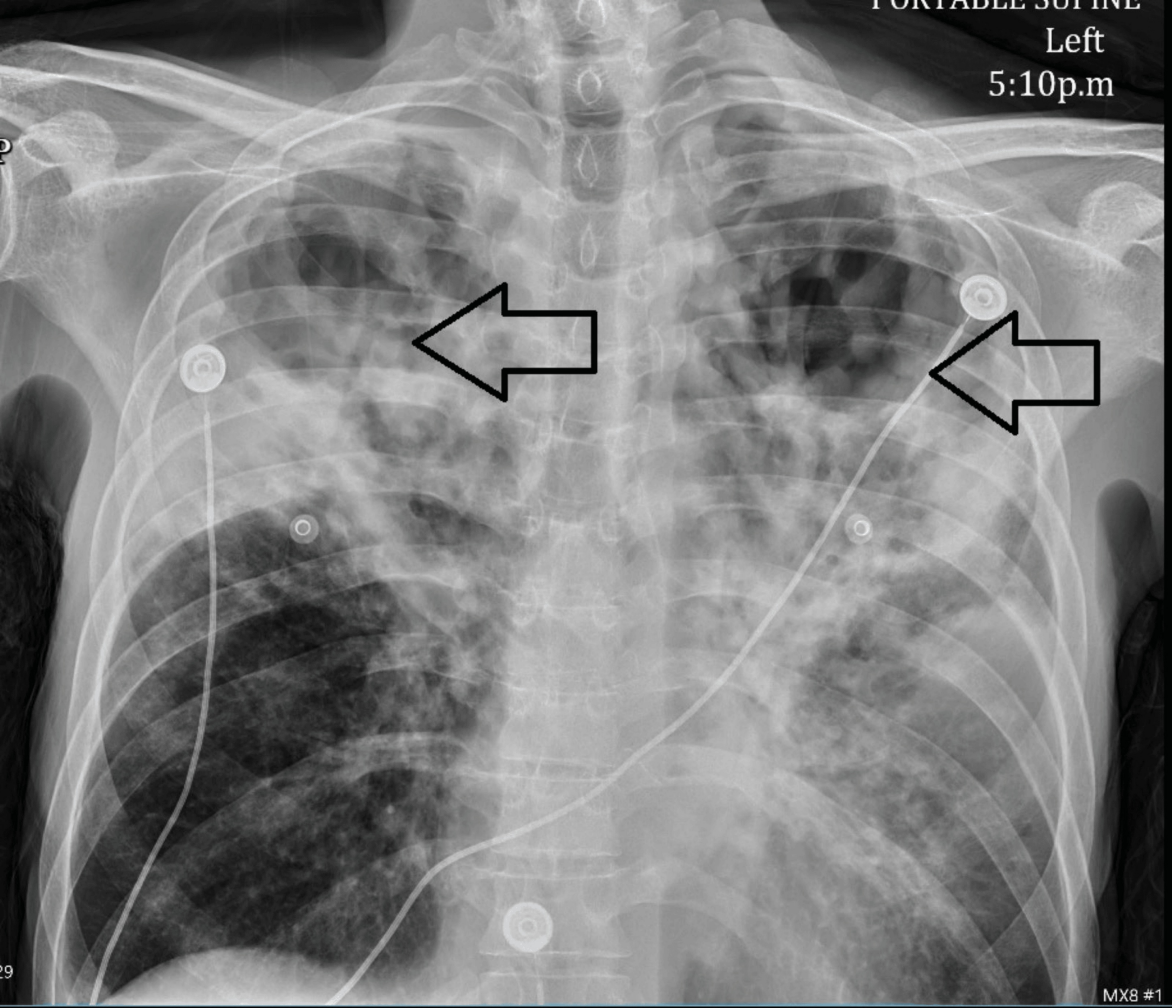
Chest X-ray showing cavitary lesions (black arrows)

Human immunodeficiency virus testing was negative, and hemoglobin A1c was 6.5% (<5.7% is considered nondiabetic), which was consistent with diabetes. QuantiFERON-TB Gold was positive. EKG was unremarkable. Blood cultures had been negative over multiple sessions. *Legionella pneumophila* in sputum and *Legionella* urine antigen were negative. The patient had persistent mild hyponatremia with hypomagnesemia requiring supplementation. Chest X-ray on admission showed heterogeneous bilateral airspace opacities and cavitary lesions in bilateral lung apices (Figure 1).

**Figure 2:**
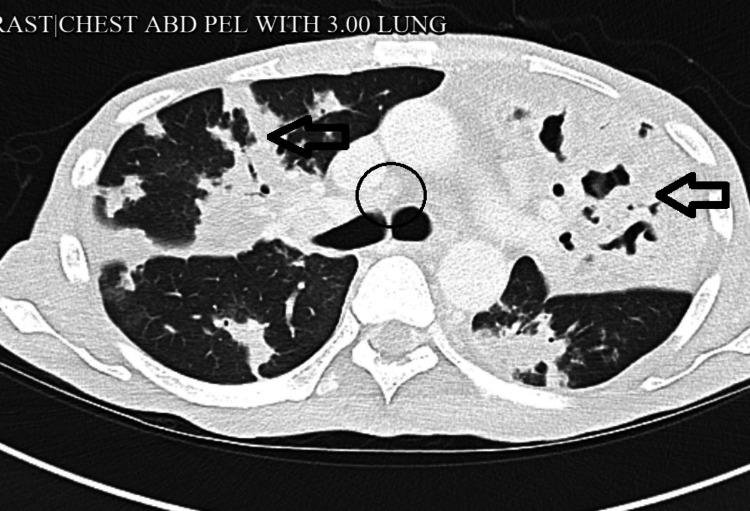
CT scan of the chest showing large bilateral upper-lobe lobulated thick-walled pulmonary cavities compatible with cavitating tuberculosis (black arrows) with lymphadenopathy (black circle) CT, computed tomography

Computed tomography (CT) of the chest demonstrated large, thick-walled cavitary lesions in both upper lobes with multifocal consolidations and bilateral hilar lymphadenopathy (Figure 2).

Initial acid-fast bacilli (AFB) smears were positive, and MTB polymerase chain reaction (PCR) confirmed smear-positive pulmonary TB, prompting initiation of standard four-drug therapy (rifampin, isoniazid, pyrazinamide, and ethambutol). Concurrently, sputum cultures revealed coinfection with *M. abscessus*, MAC, and *Klebsiella pneumoniae*. *M. abscessus* was susceptible to amikacin and showed intermediate susceptibility to cefoxitin and imipenem. Testing for the *erm(41)* gene (inducible macrolide resistance) was not performed. The diagnosis of NTM pulmonary disease was consistent with American Thoracic Society (ATS)/Infectious Diseases Society of America (IDSA) criteria, given multiple positive sputum cultures obtained on separate occasions and progressive radiologic abnormalities. Infectious disease specialists expanded therapy to include IV amikacin and cefoxitin for *M. abscessus* and oral azithromycin for MAC, while piperacillin-tazobactam was started for presumed *K. pneumoniae* pneumonia. No infusion-related adverse effects were reported. Despite broad antimicrobial therapy, serial AFB smears remained positive four months later, delaying discharge until smear conversion. Because AFB smears do not distinguish MTB from NTM, the organism responsible for persistent positivity and microbiologic conversion could not be determined. The patient required intermittent supplemental oxygen for exertional hypoxia but remained afebrile. Care continues under a coordinated multidisciplinary team involving infectious disease, pulmonology, and public health.

## Discussion

This case illustrates the complexity of concurrent pulmonary MTB and NTM infections. Coinfection of MTB and *M. abscessus*/*M.* *avium* in an immunocompetent patient is uncommon and presents diagnostic and therapeutic challenges [1,2]. Both pathogens can produce cavitary lung disease and similar constitutional symptoms (cough, weight loss, and fatigue), making initial differentiation difficult. Radiologically, the diffuse bilateral cavitary lesions and consolidations in our patient were consistent with active TB but could also be seen with NTM. Indeed, MAC infection is known to mimic TB radiographically [3-5]. In ambiguous cases, microbiological testing is essential: acid-fast smears cannot discriminate MTB from NTM, so culture and molecular identification (PCR/matrix-assisted laser desorption/ionization (MALDI)) are required [1]. In our patient, the combination of positive MTB PCR and multiple culture-confirmed NTM organisms, along with the clinical picture, supported true coinfection rather than mere NTM colonization [1].

NTM species such as MAC and *M. abscessus* increasingly cause lung infections in at-risk populations (e.g., bronchiectasis, prior TB, or immunosuppression) [3]. Interestingly, this patient had no known immunodeficiency or structural lung disease other than a mild smoking history. His pulmonary cavities from MTB may have provided a nidus for environmental NTM. Current guidelines highlight that differentiating NTM colonization from disease depends on clinical, radiological, and microbiological criteria [1]. Karki et al. noted that co-isolated NTM are often considered colonizers, but true disease must be established before treatment [1]. In our case, the persistence of productive cough, imaging findings of progressive cavitation, and repeated NTM-positive cultures supported initiating NTM-directed therapy.

Treatment of MTB-NTM coinfection must be carefully coordinated. Standard therapy for drug-sensitive TB (six months of rifampin, isoniazid, pyrazinamide, and ethambutol) remains the backbone for MTB [6]. However, NTM species generally do not respond to these drugs [3] and require specific regimens. For *M. abscessus*, current guidelines recommend macrolides (azithromycin or clarithromycin) and amikacin as cornerstones [4]. Our patient’s *M. abscessus* isolate was macrolide-susceptible; we added azithromycin and high-dose intravenous amikacin as recommended. *M. abscessus* often harbors inducible macrolide-resistance (*erm*) genes, but when absent or before resistance develops, macrolides plus companion drugs (amikacin and beta-lactams) offer the best outcome [4]. For MAC, the usual regimen includes a macrolide, rifamycin, and ethambutol, coinciding fortunately with our ongoing rifampin and azithromycin. Ethambutol was already part of the TB regimen. Thus, MTB treatment (rifampin/ethambutol) and the added NTM drugs (amikacin, cefoxitin, and azithromycin) were combined under close monitoring. This dual therapy is complex: for example, rifampin induces metabolism of macrolides, and overlapping toxicities (e.g., hepatotoxicity) must be watched.

We also addressed bacterial superinfection. *K. pneumoniae* is a known opportunistic pathogen in TB patients, especially those hospitalized or immunocompromised [6]. Coinfection with K. pneumoniae can worsen outcomes and mimic TB symptoms [6]. In line with Cioboata et al., we noted that K. pneumoniae-TB coinfection produces overlapping respiratory symptoms and occurs in patients with chronic disease [6,7]. We treated the patient’s *Klebsiella* pneumonia with piperacillin-tazobactam according to culture results.

Review of the literature shows few similar cases. Ishiekwene et al. described an immunocompetent patient with pulmonary TB and *M. abscessus* coinfection, stressing that clinicians should suspect *M. abscessus* when TB patients have atypical courses [2,8]. Karki et al. reported a 22-year-old with simultaneous pulmonary TB and MAC, noting the challenge of distinguishing colonization from disease and the need for microbiological confirmation [9,10]. Our case parallels these reports in management strategy but is notable for having both *M. abscessus* and MAC.

The patient’s prolonged smear positivity highlights another point: coinfections and extensive cavitary disease may delay sputum conversion. He remained in airborne isolation until at least three negative AFB smears could be documented (per public health guidelines). Multidisciplinary care (infectious diseases, pulmonology, and TB control) is essential, given the need for prolonged therapy, monitoring for drug resistance or toxicity, and public health oversight for TB.

## Conclusions

This case underscores the diagnostic and therapeutic complexity of pulmonary mycobacterial disease when MTB and NTM coexist, even in an immunocompetent host. Although TB remains a common global infection, increasing recognition of NTM as true pulmonary pathogens necessitates heightened clinical vigilance, particularly when patients demonstrate atypical courses, extensive cavitary disease, or delayed microbiologic response to standard therapy. Our patient illustrates how overlapping clinical symptoms, radiographic findings, and acid-fast smear positivity can obscure the presence of multiple mycobacterial species, emphasizing the importance of comprehensive microbiologic evaluation beyond initial smear and PCR testing. This case highlights several key takeaways. First, true coinfection with MTB and NTM can occur in the absence of overt immunosuppression or significant preexisting lung disease, suggesting that structural lung damage from active TB may itself predispose to secondary NTM infection. Second, distinguishing NTM colonization from active disease requires careful integration of clinical presentation, imaging progression, and repeated culture results; premature dismissal of NTM as contaminants may delay appropriate therapy. Third, management of MTB-NTM coinfection demands individualized, multidisciplinary coordination to balance effective antimicrobial coverage, drug-drug interactions, and cumulative toxicities, while maintaining adherence to public health measures for TB control. Finally, this case reinforces the need for clinicians to consider mixed mycobacterial infections in patients with persistent symptoms or delayed sputum conversion despite appropriate TB therapy. Early recognition and tailored treatment may improve clinical outcomes and reduce prolonged hospitalization and isolation. As the incidence of NTM lung disease continues to rise, awareness of MTB-NTM coinfection is essential for timely diagnosis, optimal treatment, and improved patient care.
